# Gene Expression Profile of Dendritic Cell-Tumor Cell Hybrids Determined by Microarrays and Its Implications for Cancer Immunotherapy

**DOI:** 10.1155/2015/789136

**Published:** 2015-10-28

**Authors:** Jens Dannull, Chunrui Tan, Christine Farrell, Cynthia Wang, Scott Pruitt, Smita K. Nair, Walter T. Lee

**Affiliations:** ^1^Department of Surgery, Duke University Medical Center, Durham, NC 27710, USA; ^2^Department of Surgery, Division of Otolaryngology-Head and Neck Surgery, Duke University Medical Center, Durham, NC 27710, USA; ^3^Duke University, Durham, NC 27710, USA; ^4^Duke University School of Medicine, Durham, NC 27710, USA; ^5^Division of Experimental Medicine, Merck, Rahway, NJ 07065, USA; ^6^Durham VA Medical Center, Section of Otolaryngology-Head and Neck Surgery, Durham, NC 27705, USA

## Abstract

*Background*. Dendritic cell- (DC-) tumor fusion cells stimulate effective* in vivo* antitumor responses. However, therapeutic approaches are dependent upon the coadministration of exogenous 3rd signals. The purpose of this study was to determine the mechanisms for inadequate 3rd signaling by electrofused DC-tumor cell hybrids.* Methods*. Murine melanoma cells were fused with DCs derived from C57BL/6 mice. Quantitative real-time PCR (qPCR) was used to determine relative changes in Th (T helper) 1 and Th2 cytokine gene expression. In addition, changes in gene expression of fusion cells were determined by microarray. Last, cytokine secretion by fusion cells upon inhibition of signaling pathways was analyzed by ELISA.* Results*. qPCR analyses revealed that fusion cells exhibited a downregulation of Th1 associated cytokines IL-12 and IL-15 and an upregulation of the Th2 cytokine IL-4. Microarray studies further showed that the expression of chemokines, costimulatory molecules, and matrix-metalloproteinases was deregulated in fusion cells. Lastly, inhibitor studies demonstrate that inhibition of the PI3K/Akt/mTOR signaling pathway could restore the secretion of bioactive IL-12p70 by fusion cells. *Conclusion*. Our results suggest that combining fusion cell-based vaccination with administration of inhibitors of the PI3K/Akt/mTOR signaling pathway may enhance antitumor responses in patients.

## 1. Introduction

Dendritic cells (DCs) have been identified as a key component in manipulating and stimulating the immune system [1]. Activated DCs are potent antigen presenting cells that express both major histocompatibility complex (MHC) class I and II molecules (Signal 1) and costimulatory molecules (Signal 2) and secrete immune modulating cytokines (Signal 3) resulting in activation of T lymphocytes [2]. Depending on the cytokine environment, DCs may elicit either a Th (T helper) 1 or Th2 CD4 T-cell response. For tumor immunotherapy, induction of a Th1 T-cell response is pivotal, and secretion of IL-12 (interleukin 12) by DCs is of critical importance for differentiation of naive T cells into Th1 cells [3]. Furthermore, IL-12 stimulates the production of interferon-gamma (IFN-*γ*) and tumor necrosis factor-alpha (TNF-*α*) from T cells and natural killer cells. In contrast, Th2 responses, associated with cytokines IL-4, IL-5, IL-6, and IL-10, suppress Th1 activity and may anergize effector T cells to tumor antigens [4].

DCs are the basis for numerous immunotherapy strategies against a variety of cancers [5]. One of these strategies involves fusing DCs with tumor cells using electrical currents in a method called electrofusion, hence combining the antigen presenting properties of DCs with the full repertoire of antigens present within a tumor cell in order to stimulate effector T cells [6, 7]. While DC-tumor hybrids alone are insufficient to elicit significant immune responses* in vivo* and are critically dependent upon exogenously administered 3rd signal adjuvants, murine studies using DC-tumor hybrids for vaccination given concomitantly with an adjuvant third signal, such as IL-12, OX-40-, 4-1BB-monoclonal antibody, or toll-like receptor agonists, showed regression of tumor metastases after a single vaccination in several tumor types including melanoma, breast, sarcoma, and squamous cell carcinoma [8–11]. However, systemic delivery of 3rd signal along with a DC-tumor fusion vaccine is clinically problematic due to 3rd signal toxicity and/or availability [12]. Therefore, a better understanding of the mechanisms affecting the dependence of DC-tumor fusions on 3rd signal adjuvants is of paramount importance for optimizing this immunotherapeutic approach.

In this study, we show that production of the Th1 skewing cytokine IL-12 was dramatically downregulated in DC-tumor fusion cells. Microarray analyses further reveal changes in chemokine production and expression of costimulatory molecules. In addition, gene products that are involved in signaling pathways including NF*κ*B (nuclear factor kappa-light-chain-enhancer of activated B-cells), PI3K/Akt/mTOR (phosphatidylinositol 3-kinase/Akt, protein kinase B/mammalian target of rapamycin), Wnt (wingless-related integration site), and MAPK (mitogen-activated protein kinase) were differentially expressed in fusion cells. Inhibitor studies revealed that interruption of the canonical Wnt pathway did not affect IL-12 production by DC-tumor fusion cells and that inhibition of MEK (mitogen extracellular signal-regulated kinase) only increased IL-12 production marginally. In contrast, IL-12 production could significantly be enhanced by treatment of DC-tumor hybrids with inhibitors of the PI3K and mTOR. Given the critical role of the PI3K/Akt/mTOR signaling pathway in cancer biology and the immunostimulatory effect of PI3K/Akt/mTOR inhibitors on DC-tumor hybrids, combination therapy may represent a promising and novel cancer vaccine with enhanced clinical impact.

## 2. Materials and Methods

### 2.1. Mice

Female C57BL/6 mice were purchased from Charles River Laboratories (Raleigh, NC). The mice were maintained in a specific pathogen-free environment. All mice were used at 8 to 12 weeks of age. Animals were housed in a specific pathogen-free environment at the animal facility of the Durham Veteran Affairs Medical Center. All mice used in this study were cared for in accordance with the Guide for Humane care and use of Laboratory Animals published by the National Institutes of Health. All the animal experimental protocols were approved by the Duke University Medical Center Institutional Animal Care and Use Committee.

### 2.2. Tumor Cell Lines

D5LacZ is a *β*-galactosidase expressing derivative of the B16 F10.9 melanoma cell line and has been shown to be poorly immunogenic. Its fusion parameters as well as* in vivo* characteristics have been well studied [13]. Cells were cultured in complete media (CM) composed of RPMI 1640 media supplemented with 10% fetal bovine serum, 2 mM L-glutamine, 0.1 mM nonessential amino acids, 1 mM sodium pyruvate, 100 U/mL penicillin, 100 *μ*g/mL streptomycin, 0.5 *μ*g/mL fungizone, 50 *μ*g/mL gentamicin, and 5 × 10^−5^ M 2-mercaptoethanol (Invitrogen, Carlsbad, CA). These cells were maintained at 37°C with 5% CO_2_, harvested following a short incubation period with 0.05% trypsin with EDTA, and irradiated at 100 Gy prior to use.

### 2.3. DC Generation

DCs were generated from femoral and tibial bone marrow cells of C57BL/6 mice. Erythrocytes were lysed with ACK lysis buffer. B- and T-lymphocytes were depleted using antibody-coated magnetic beads (Dynal Biotech, Carlsbad, CA). The DC-enriched cell fraction was then cultured in CM supplemented with 10 ng/mL GM-CSF and 10 ng/mL IL-4 (Peprotech, Rocky Hill, NJ) at a concentration of 0.5 × 10^6^ cells/mL at 37°C with 5% CO_2_. On day 6, cells were harvested, resuspended in fresh CM + GM-CSF/IL-4 media at 1 × 10^6^ cells/mL, and incubated at 37°C with 5% CO_2_ for 24 hours. Then, LPS (lipopolysaccharide, 100 ng/mL, Sigma-Aldrich, Saint Louis, MO) was added to stimulate DC maturation. After 24 hours, FACS analysis was used to confirm mature DC phenotype as previously described [14]. After 24 hours, DCs were stained intracellularly with CFSE prior to use (Molecular Probes, Eugene, OR).

### 2.4. Electrofusion of DCs and Tumor Cells

Irradiated tumor cells and CFSE stained DC were mixed in a 1 : 1 ratio and washed in prefusion media, followed by resuspension in fusion media at a concentration of 20 × 10^6^ cells/mL. For electrofusion, the pulse generator (model ECM 2001 generator, BTX Instruments, San Diego, CA) was used. Cells were exposed to two consecutive, independent electrical currents: (1) a low voltage alternating current of 120 V/cm for 10 seconds to achieve alignment and chain formation, and (2) a high voltage direct current of 1100 V/cm for 25 microseconds to cause a reversible breakdown of cell membranes. The multinucleated hybrid cells were allowed to stand for at least 5 minutes before incubation in culture media overnight at 37°C with 5% CO_2_.

### 2.5. Cell Sorting

To separate unfused tumor cells (T) from T-T hybrids and unfused DCs from DC-DC hybrids FACS sorting by size on forward scatter (FSC) and side scatter (SSC) was employed (data not shown). All cells larger than the unfused cell populations were considered fusion hybrids. DC-T hybrids were purified using a combination of mechanical and FACS sorting techniques, based on their plastic adherence characteristics as well as CFSE staining. Tumor cells are adherent, while DCs are nonadherent. Therefore, after electrofusion and overnight culture, the nonadherent cell population representing unfused DCs and DC-DC hybrids was discarded. FACS was then performed only on the adherent cell population containing unfused tumor cells, T-T hybrids, and DC-T hybrids. Since only DCs were stained with CFSE, FACS sorting was used to separate CFSE positive cells from the CFSE negative populations (unfused tumor cells and T-T hybrids). All cell samples were analyzed using the FACS Aria II (BD Biosciences, San Jose, CA).

### 2.6. Quantitative Real-Time PCR

24 hours after electrofusion, total RNA was isolated using the RNeasy Plus Mini Kit protocol (Qiagen, Valencia, CA). The cDNA template was synthesized from 0.5–1.0 ug of total RNA using the RT^2^ First Strand Kit protocol (SABiosciences, Frederick, MD). Each template was then combined with RT^2^ SYBR Green qPCR Master Mix (SA Biosciences) and aliquoted into a 96-well mouse common cytokine plate array (SA Biosciences). The PCR cycling program was 95°C for 10 minutes, followed by 40 cycles of 95°C for 15 seconds, and then 60°C for 1 minute on a Stratagene Mx3005p qPCR machine. Analysis of qPCR data was calculated using the ΔΔCt method. Quality control guidelines were followed according to the RT^2^ Profiler PCR Array System manual. Briefly, all threshold values (Ct) reported as greater than 35 indicated no detectable gene expression. Genomic DNA (GDNA) contamination was detected if the GDNA control Ct value was below 35. A Reverse Transcription Control (RTC) detected impurities in the RNA sample that affect the reverse transcription of the template and was considered positive if the Ct was greater than 5. qPCR data that did not meet quality control guidelines were excluded. All samples were run in duplicate, compared for consistency, and averaged. Gene expression associated with Th1 (IFN-*γ*, IL-2, IL-12p40, IL-15, IL-18, and TNF-*α*) and Th2 (IL-4, IL-10, IL-13, and IL-25) immune responses was analyzed.

### 2.7. Microarray Analysis

Total RNA was isolated from tumor cells, DCs, and DC-T fusion cells using the RNeasy Plus Mini Kit protocol (Qiagen, Valencia, CA). RNA isolation for tumor and dendritic cells was done in triplicate. For the DC-T fusion cells, RNA was extracted for each of the triplicate fusion batches. Quality check was done on a Nanodrop spectrophotometer. Triplicate samples of D5lacZ tumor cells, DCs, and DC-T fusion cells were each run through a microarray chip (Affymetrix) by the Duke DNA Microarray Core Facility. Partek Genomics Suite 6.4 (Partek Inc., St. Louis, MO) was used to perform data analysis. Robust multichip analysis (RMA) normalization was done on the entire data set. Multiway ANOVA was performed and fold change was determined to select target genes that were differentially expressed between fusion cells and DCs, or fusions cells and tumors cells, respectively. Top differentially expressed genes were selected with *p* value cutoff of 0.01 based on ANOVA test and fold change cutoff of >5. Hierarchical clustering was performed on differentially expressed genes based on Average Linkage with Pearson's Dissimilarity. Data was also analyzed by pathway using Metacore from Genego. Microarray data was analyzed on Excel and Metacore from Genego.

### 2.8. ELISA

The murine IL-4 ELISA kit (eBioscience, San Diego, CA) and the murine IL-12p70 ELISA kit (BD Biosciences, San Jose, CA) were used according to the manual provided by the manufacturer. To determine cytokine secretion by DCs or DT-tumor fusion cells, 2 × 10^5^ cells in 1 mL of AIMV media (Invitrogen, Carlsbad, CA) were incubated in the presence of 100 ng/mL of LPS for 24 hours at 37°C, 5% CO_2_. Where indicated, LPS stimulation was performed in the presence of the following inhibitors (purchased from Sigma-Aldrich, Saint Louis, MO): U0126 (1,4-diamino-2,3-dicyano-1,4-bis[2-aminophenylthio] butadiene) is a highly selective inhibitor of both MEK1 and MEK2 and was used at a concentration of 100 nM, JW 74 (4-[4-(4-methoxyphenyl)-5-[[[3-(4-methylphenyl)-1,2,4-oxadiazol-5-yl]methyl]thio]-4H-1,2,4-triazol-3-yl]-pyridine) an inhibitor of the canonical Wnt pathway was used at a concentration of 10 *μ*M, rapamycin (23,27-epoxy-3H-pyrido[2,1-c][1,4]oxaazacyclohentriacontine) forms a complex with FKBP12 (FK506 binding protein 12) that binds to and inhibits mTOR which was used at 0.5 *μ*M, and Wortmannin which inhibits the PI3K/Akt signal transduction cascade was used at 100 nM. Experiments were performed in duplicate and error bars represent the SEM (standard error of the mean).

## 3. Results

### 3.1. The Impact of DC-Tumor Fusion on Cytokine Gene Expression

**Figure 1:**
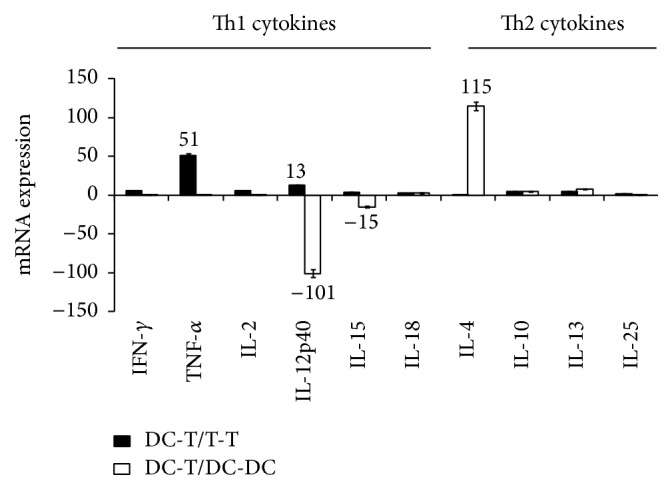
cDNAs generated from DC-DC hybrid cells (DC-DC), D5lacZ tumor cell hybrids (T-T), and DC-tumor cell hybrids were subjected to quantitative real-time PCR analyses using primer pairs which detect the Th1 cytokines IFN-*γ*, TNF-*α*, IL-2, IL-12p40, IL-15, and IL18 and the Th2 cytokines IL-4, IL-10, IL-13, and IL-25. Samples were run in duplicate and difference in mRNA expression was calculated using the ΔΔCt method. Error bars represent the standard error of the mean. One representative experiment out of three independent experiments is shown.

In a first set of experiments, D5LacZ tumor-tumor (T-T) cell hybrids, DC-DC hybrids, and DC-T hybrids were generated by electrofusion. Fusion cells were purified by FACS and RNA isolated from hybrid cells was analyzed by quantitative real-time PCR (qPCR) for expression levels of mRNAs encoding the Th1 cytokines IFN-*γ*, TNF-*α*, IL-2, IL-12p40 (the *β*-subunit of bioactive IL-12p70), IL-15, and IL-18 or the Th2 cytokines IL-4, IL-10, IL-13, and IL-25.  Figure 1 shows the results of our qPCR analyses. Comparison of DC-T hybrids with DC-DC fusion cells (white bars) reveals that cytokines associated with a Th1 response including IL-12p40 and IL-15 were downregulated by more than 100- and 15-fold, respectively. In contrast, the Th2 cytokine IL-4 was dramatically upregulated by 115-fold. Among all cytokines analyzed, only TNF-*α* and IL-12p40 exhibited higher expression levels in DC-T fusion cells when compared to T-T fusions (Figure 1, black bars).

In another series of experiments, the Th1 and Th2 cytokine expression profile of cells exposed to electrofusion was compared to unexposed cells. However, no significant changes in cytokine gene expression between tumor cells and T-T fusion cells or DCs and DC-DC fusion cells were observed (data not shown). For this reason, we focused on the comparison of gene expression levels between DC-T hybrid cells and DCs in the subsequent analyses presented in this study.

### 3.2. Microarray: Cytokines and Cytokine Receptors

**Figure 2:**
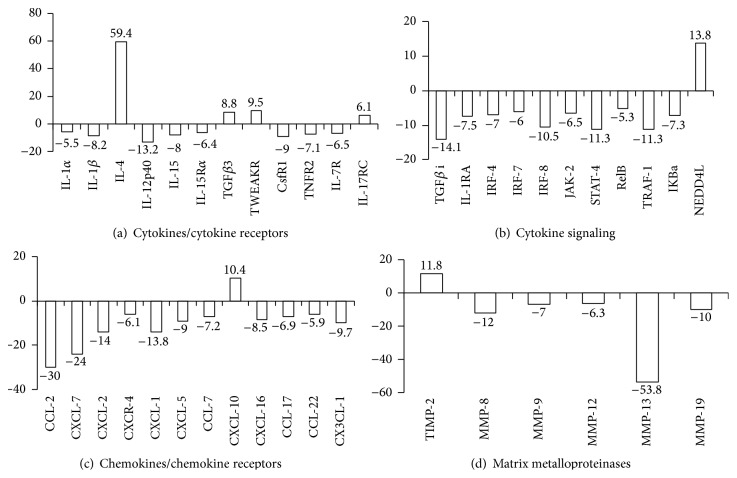
Expression profile of cytokines and cytokine receptors. (a) Molecules involved in cytokine signaling, (b) chemokines and chemokine receptors, and (c) matrix metalloproteinases (d). Data from microarrays are presented and fold differences in expression between DC-tumor cell hybrids and DCs are shown. Positive values indicate upregulation in DC-tumor cell hybrids, while negative values indicate downregulation. When expression levels from different probe sets were available for the same gene product (IL-12p40, CsfR1, TGF*β*i, IL-1RA, and TIMP-2), the average of their values was presented.

**Figure 3:**
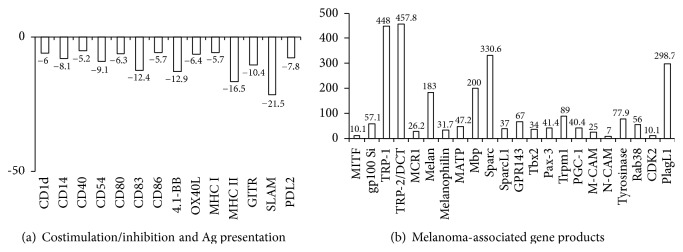
Expression level of costimulatory molecules and molecules involved in antigen (Ag) presentation (a) or melanoma-associated gene products (b) in DC-tumor cell hybrids. Microarray data for DC-tumor cell hybrids and DCs were compared. Positive values indicate upregulation in DC-tumor cell hybrids. Data from different probe sets were obtained for CD1d, HLA-class II (HLA-Q6 and HLA-Q7), MITF, Melanophilin, Pax3, PGC-1, MATF, SPARC, Mbp, Tyrosinase, Trp1, and Trp2. For these gene products the average in gene expression is shown.

We next sought to determine changes in the expression of genes that may negatively impact the immunologic properties of DC-T fusion cells. In order to do so, RNAs were isolated from FACS-isolated DC-T hybrids cells, DCs, or D5LacZ tumor cells, and microarray assays were performed. Consistent with our qPCR data, expression of IL12p40 and IL-15 by DC-T fusion cells was markedly downregulated (13.2- and 8-fold) when compared to DCs, albeit to a lesser degree than observed in PCR analyses (Figure 2(a)). Also, IL-4 was upregulated 59.4-fold in DC-T fusions. The proinflammatory cytokines IL-1*α* and IL-1*β* were downregulated 5.5- and 8.2-fold, respectively, while TGF*β*3 was upregulated 8.8-fold. Furthermore, we observed a downregulation of receptors for colony-stimulating factor (CsfR1), TNF-*α* (TNFR2), and IL-7 (IL-7R). In contrast the receptors for TWEAK (TNF-like weak inducer of apoptosis, TWEAKR) and for IL-17 (IL-17RC) were upregulated 9.5- and 6.5-fold. While overexpression of IL-17RC has been implicated in Bcl-2- and Bcl-X_L_-independent protection of cancer cell lines from TNF*α*-induced apoptosis [15], TWEAKR signaling has been shown to enhance the expression of NF*κ*B (nuclear factor kappa-light-chain-enhancer of activated B-cells)-regulated genes including IL-6, IL-8, RANTES, and ICAM-1 (CD54) [16]. However, upregulation of none of these gene products was observed in our study (Figures 2(a), 2(c), and 3(a)).

### 3.3. Microarray: Gene Products Involved in Cytokine Signaling

In addition to cytokine gene and cytokine receptor expression, there were also significant changes in the expression level of gene products that are involved in cytokine signaling (Figure 2(b)). Expression of TGF*β*i (transforming growth factor beta-induced), a protein that is induced by TGF*β* and that acts to inhibit cell adhesion [17], was downregulated 14.1-fold. Downregulation of this gene product was unexpected given that TGF*β*3 was upregulated in DC-T hybrids (Figure 2(a)) and implies that the TGF*β*-signaling pathway may not be hyperactive in DC-T hybrid cells. There were no differences in expression levels of TGF*β*-receptors between DCs and DC-T hybrid cells. However, NEDD4L (neural precursor cell expressed developmentally downregulated gene 4-like) was upregulated 13.8-fold in DC-T hybrid cells. NEDD4L negatively regulates TGF*β* signaling by ubiquitination-mediated degradation of TGF-*β* receptor 1 and receptor-regulated Smad2 (mothers against decapentaplegic homolog 2) [18]. As such, it is reasonable to assume that NEDDL4 overexpression suppressed transcriptional activity induced by TGF*β*.

Expression of IL-1RA, the interleukin-1 receptor antagonist, which modulates a variety of IL-1 related immune and inflammatory responses, was downregulated 7.5-fold. Moreover, expression of IRFs (interferon regulatory factors) 4, 7, and 9, which are involved in transcriptional regulation of type I interferon genes, interferon signaling, and hence the Janus kinase- (JAK-) Signal Transducer and Activator of Transcription (STAT) pathway [19], was downregulated 7-, 6-, and 10.5-fold, respectively. Also, JAK-2 and STAT-4, known to be involved in IL-12 receptor signaling [20], are downregulated 6.5- and 11.3-fold.

Last, three gene products that are associated with NF-*κ*B signaling were found to be downregulated in DC-T hybrid cells, namely, RelB (reticuloendotheliosis viral oncogene homolog B, 5.3-fold), TRAF-1 (TNF receptor associated factor-1, 11.3-fold), and the NF-*κ*B inhibitor I*κ*B*α* (nuclear factor of kappa light polypeptide gene enhancer in B-cells inhibitor alpha, 7.3-fold).

RelB is known to form heterodimers with NF-*κ*B p50 or p52 [21], and TRAF-1 forms a heterodimeric complex with TRAF2, which is required for TNF-*α*-mediated activation of MAPK8/JNK (Jun kinase) and NF-*κ*B. On the other hand, the inhibitor of NF-*κ*B [22], I*κ*B*α*, is also downregulated. These results are somewhat contradictory and argue that NF-*κ*B activity in DC-tumor fusion cells is not regulated at the transcriptional level.

### 3.4. Microarray Analyses: Chemokines and Chemokine Receptors

As shown in Figure 2(c), expression of chemokines or their receptors which are involved in chemotaxis of neutrophils, monocytes, DCs, T cells, and NK cells were generally downregulated in DC-tumor fusions, with the exception of CXCL-10 (IP-10, interferon-gamma-induced protein 10). Surprisingly, even chemokines involved in chemotaxis of Th2 cells and regulatory T cells (CCL-17 and CCL-22) were downregulated while IP-10 which is implicated in the induction of Th1 responses and chemotaxis of Th1 cells was significantly upregulated [23, 24]. We therefore hypothesize that the chemokine expression profile of DC-tumor hybrids does not have a major impact on the Th-polarizing capacity of DC-tumor hybrid cells.

### 3.5. Microarray Analyses: Matrix Metalloproteinases (MMPs)

It has been demonstrated that the expression of matrix metalloproteinases MT-1 (MMP-14) and MMP-9 is a major contributing factor to the migratory capacity of DCs to lymph nodes through the degradation of extracellular matrix components. In this context, MMP-9 activity is of particular importance since it cleaves collagen IV, a major component of basement membranes. Furthermore, it has been shown that the balance of MMP-9 and TIMP (tissue inhibitor of MMPs) expression is crucial for DC migration* in vivo* [25].

Our data reveal that TIMP-2 was upregulated 11.8-fold in DC-T fusion cells, while MMP-9 is downregulated 7-fold (Figure 2(d)). As such, these results suggest that the migratory capacity of DC-T hybrids toward lymph-node derived chemokines, namely, CCL-19 and CCL-21, may be impaired.

### 3.6. Microarray Analyses: Costimulatory Molecules and Antigen Presentation

As shown in Figure 3(a), expression of genes involved in antigen presentation in the context of MHC classes I and II or Cd1d was downregulated 5.7-, 16.5-, and 6-fold in fusion cells. Furthermore, the expression of all well-established costimulatory molecules, including CD40, CD54, CD80, CD83, CD86, 4-1BB, GITR (glucocorticoid-induced TNFR-related protein), OX40L, and SLAM (signaling lymphocytic activation molecule), was downregulated in DC-tumor fusion cells. These data explain to some degree why targeting of costimulatory molecules with agonistic antibodies can enhance the potency of DC-tumor fusion-based vaccines, as has been described previously.

Last, expression of PD-L2 (programmed death ligand 2), an inhibitory immune checkpoint molecule, was suppressed 7.8-fold in DC-fusion cells. No differences in PD-L1 expression between DCs and DC-T hybrid cells were observed.

#### 3.6.1. Microarray Analyses: Melanoma-Associated Gene Products

The development of melanocytes is highly dependent on the action of the microphthalmia-associated transcription factor (MITF) which has been shown to regulate a broad variety of genes, whose functions range from pigment production to cell-cycle regulation, migration, and survival [26]. MITF was upregulated in DC-tumor fusion cells (Figure 3(b)). Concomitantly, also MITF-regulated mRNAs encoding melanoma antigens, including Tyr (Tyrosinase), TRP-1 and TRP-2 (Tyrosinase-related protein), gp100 (Silver), Melan, Melanophilin, M-CAM (melanoma cell adhesion molecule), and MATP (membrane-associated transporter protein also known as solute carrier family 45 member 2 (SLC45A2) or melanoma antigen AIM1), were also highly upregulated. Moreover, expression of MITF-regulated MCR1 (melanocortin 1 receptor), TRPM1 (transient receptor potential cation channel subfamily M member 1), GPR143 (G protein-coupled receptor 143), and Mbp (myelin basic protein) was highly upregulated in fusion cells. Expression of Mbp by melanoma cells is somewhat surprising, but it has been shown that B16F10 cells undergo differentiation to a myelinating glial phenotype characterized by induction of the transcriptional activity of the MBP promoter [27].

Last, Osteonectin (secreted protein acidic and rich in cysteine (SPARC)), which has been implicated in metastasis of melanoma to the lungs [28], and PlagL1 (Pleomorphic adenoma gene-like 1), a potential tumor suppressor gene [29], were also overexpressed in DC-tumor fusions. These results suggest that the entire antigenic repertoire of melanoma cells is indeed strongly expressed in DC-tumor hybrid cells, as has been hypothesized.

#### 3.6.2. Microarray Analyses: Signal Transduction Pathways

**Figure 4:**
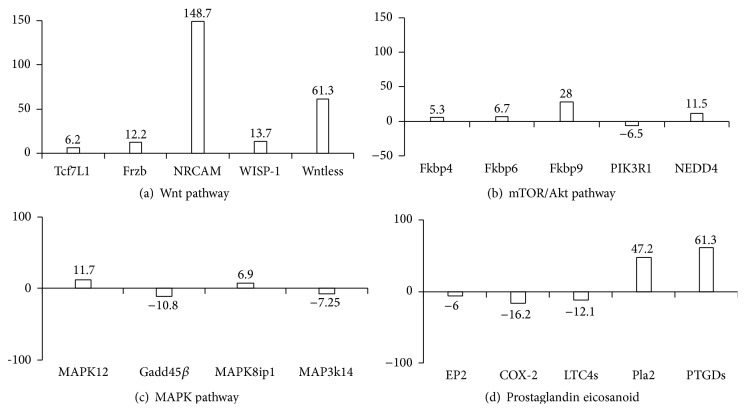
Expression level of molecules involved in signal transduction of the Wnt pathway (a), the mTOR/Akt pathway (b), the MAPK pathway (c), or prostaglandin, eicosanoid pathway (d). Microarray data for DC-tumor cell hybrids and DCs were compared. Positive values indicate upregulation in DC-tumor cell hybrids. Data from different probe sets were obtained for Frzb, WISP-1, and Gadd45*β*. For these gene products the average in gene expression is shown.

Next, we analyzed expression levels of genes that are involved in signaling pathways known to be aberrantly regulated in cancer cells [30]. The transcription factor Tcf7L1 (transcription factor 7-like 1) which is activated by *β*-catenin and thus mediated Wnt signaling was upregulated 6.2-fold (Figure 4(a)). Also, expression of Frzb (Frisbee), a Wnt-binding protein and competitor for the cell-surface receptor Frizzled, and expression of Wntless (G protein-coupled receptor 177), another receptor for Wnt proteins, were increased 12.2- and 61.3-fold. Furthermore, target genes of the canonical Wnt pathway, WISP-1 (WNT1-inducible-signaling pathway protein 1) [31] and NRCAM (neuronal cell adhesion molecule) [32], were upregulated 13.7- and 148.7-fold, indicating activation of the Wnt pathway in DC-tumor fusion cells.

Expression of the FK506 binding proteins FKBP4, FKBP6, and FKBP9, immunophilins known to interact with mTOR [33], was upregulated in DC-tumor fusions 5.3-, 6.7-, and 28-fold (Figure 4(b)). Furthermore, expression of PIK3R1 (phosphatidylinositol 3-kinase regulatory subunit alpha, p85*α*) was downregulated 6.5-fold, which may indicate aberrant activity of PI3K in DC-tumor fusion cells. In addition, we observed that NEDD4 was upregulated 11.5-fold. NEDD4 directly binds to and poly-ubiquitinates PTEN (phosphatase and tensin homolog), targeting it for proteasomal degradation [18]. PTEN is a tumor suppressor that negatively regulates the PI3K/Akt pathway. Therefore, posttranslational suppression of its expression level may lead to hyperactivation of the PI3K/Akt signaling pathway [18].

The LPS-inducible mitogen-activated protein kinase 12 (MAPK12), also known as extracellular signal-regulated kinase 6 (ERK6) or p38-*γ*, was upregulated 11.7-fold in DC-tumor fusions (Figure 4(c)). In contrast, the LPS-inducible Gadd45*β* (growth arrest and DNA damage-inducible 45) was downregulated 10.8-fold. Gadd45*β* is an NF-*κ*B target gene which, in combination with MEKK4, activates p38MAPK [34]. Furthermore, mitogen-activated protein kinase kinase kinase 14 (MAP3K14) also known as NF-kappa-B-inducing kinase was downregulated 7.25-fold in fusion cells. This kinase is known to bind to TRAF2 and to stimulate NF-*κ*B activity [35]. Lastly, expression of C-jun amino-terminal kinase interacting protein 1 (MAPK8ip1), a negative regulator of MAPK8 (c-jun amino-terminal kinase) [36], was upregulated 6.9-fold in DC-tumor hybrid cells.

Lipid mediators such as prostaglandins have been implicated in tumor-mediated immunosuppression [37, 38]. As presented in Figure 4(d), several genes involved in eicosanoid biosynthesis and signaling were differentially expressed in DC-tumor fusion cells. The prostaglandin E2 receptor 2 (EP2) was downregulated 6-fold. Additionally, cyclooxygenase-2 was downregulated 16.2-fold and the cysteinyl-leukotriene C4 synthase (LTC4s) was downregulated 12.1-fold. In contrast, phospholipase A2 (Pla2) and prostaglandin D2 synthase (PGDs) were upregulated 47.2- and 61.3-fold, respectively.

### 3.7. Inhibitor Studies

We next sought to determine whether inhibition of signaling pathways, for which inhibitors are available clinically, could restore secretion of bioactive IL-12p70 by DC-tumor fusion cells. We chose Wortmannin as an inhibitor of PI3K upstream of Akt (PKB), U0126 as an inhibitor of MEK1 and MEK2, rapamycin as an inhibitor of mTOR, and JW74 as an inhibitor of the canonical Wnt pathway. Admittedly, our data provide several lines of evidence that NF-*κ*B-signaling is impaired in DC-tumor fusion cells, but, even though NF-*κ*B-inhibitors are starting to emerge in the clinic, it would obviously not make sense to administer NF-*κ*B-agonists to cancer patients. We therefore omitted stimulators of NF-*κ*B-activity in our assay.

**Figure 5:**
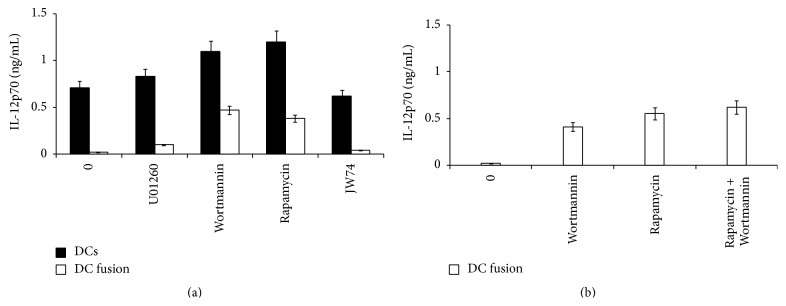
(a) Secretion of IL-12p70 by DCs and DC-tumor cell hybrids in the presence of signal transduction inhibitors. DCs (black bars) or DC-tumor cell hybrids (white bars) were stimulated with LPS and supernatants were analyzed by ELISA as described in Section 2. Stimulations were performed in the absence of inhibitor (0), or in the presence of MEK inhibitor (U0126), PI3K inhibitor Wortmannin, mTOR inhibitor rapamycin, or JW74, an inhibitor of the canonical Wnt pathway. Experiments were performed in duplicate and the standard error of the mean is presented. (b) Secretion of IL-12p70 DC-tumor cell hybrids. DC-tumor cell hybrids were stimulated with LPS in the presence or absence of inhibitors as indicated. Experiments were performed in duplicate and the standard error of the mean is presented.

DCs and DC-tumor fusion cells were stimulated with LPS in the presence or absence of inhibitors as indicated in Figure 5(a) and supernatants were analyzed for IL-12p70 secretion by ELISA. As expected, DC-tumor fusions did not produce IL-12p70 in response, while DCs responded to LPS stimulation. U0126 led to a modest increase of IL-12p70 by both DC-tumor fusions and DCs. Inhibition of PI3K with Wortmannin and inhibition of mTOR with rapamycin increased secretion of IL12-p70 significantly (11-13-fold). Inhibition of the canonical Wnt pathway with JW74 did not have any impact on IL-12p70 production by DCs or DC-tumor fusion cells. We next asked whether combined inhibition of PI3K and of mTOR could further enhance IL-12p70 secretion by DC-tumor fusion cells. As shown in Figure 5(b), combining Wortmannin and rapamycin to inhibit PI3K and mTOR did not significantly enhance IL-12p70 secretion by DC-tumor hybrid cells, hence excluding a synergistic or additive effect of these inhibitors.

## 4. Discussion 

This study is the first to investigate the mechanisms responsible for the dependence of DC-tumor hybrid vaccines on exogenously provided 3rd signal adjuvants. Several hypotheses have been postulated regarding tumor cell-mediated inhibition of immune responses. These include the induction of apoptosis of immune cells via expression of Fas ligand, TRAIL (TNF-related apoptosis-inducing ligand) [39, 40], or PD-L1 and PD-L2 (programmed death ligand) [41]. Furthermore, induction of tolerance through cytokines such as TGF-*β*, IL-6, and IL-10 [42] or lipid mediators [37, 38] has been described. Lastly, activation of the MAPK pathway by melanoma cells has been described as a mechanism to inhibit IL-12 production by DCs in a paracrine manner [43]. Our results do not provide evidence for overexpression of apoptosis-inducing ligands by DC-tumor cell hybrids, nor did we observe an enhanced production of tolerance-inducing cytokine IL-6 or IL-10 by these cells. TGF-*β*3 was upregulated 8.8-fold in DC-tumor fusions, but the observed downregulation of the TGF*β*-induced protein in combination with upregulation of NEDD4L argues against a major impact of this cytokine on fusion cells.

Surprisingly, despite a profound upregulation of mRNA encoding IL-4 in DC-tumor hybrids, there was no evidence of IL-4 signaling in these cells. We did not observe any upregulation of target genes of the IL-4 receptor I or II, including SOCS-1, IL-4 receptor *α*, CCL11 (eotaxin 1), or Fc*ε* receptor II. In addition, there were no changes in expression of gene products that are components of the IL-4 receptors, namely, IL-4 receptor *α*, IL13 receptor *α*, and CD25.

Even though the expression levels of phospholipases A2, which release arachidonic acid from phospholipids, and of prostaglandin D2 synthase were upregulated in DC-tumor fusions, expression of cyclooxygenase, which catalyzes the downstream conversion of arachidonic acid into eicosanoids, was downregulated. Furthermore, while PGD-2 has been shown to upregulate CD80 and to downregulate IP-10 in LPS-matured DCs [44], the exact opposite was observed in our experiments (Figures 2(c) and 3(a)). Accordingly, we conclude that PGD-2 may not be the main culprit for the dramatic downregulation of IL-12 production in DC-tumor hybrids.

Dysregulation of the MAPK pathway in melanoma cells has been extensively investigated [45]. However, it has been described that, in the spontaneous B16F10 melanoma cell line, expression of p16Ink4a (inhibitor of CDK4a), which inhibits cell-cycle progression by inactivating cyclin-dependent kinases, and of p19Arf (alternate reading frame tumor suppressor), which causes Mdm2 (mouse double minute 2 homologue) induced translational silencing and p53 degradation, is lost and that there is no evidence of activation of the MAPK-signaling pathway in this cell line [46]. It is therefore highly unlikely that the MAPK-signaling pathway would be a major contributor to the loss of immunostimulatory capacity of DC-tumor hybrid cells. Nevertheless, our data reveal that treatment of DC-tumor hybrid cells with MEK inhibitor U0126 led to a modest increase in IL-12 secretion. This however might be a result of the previously published observation that treatment with U0126 can result in a slight but significant inhibition of p70^S6K^ (S6 ribosomal protein kinase) activation, a downstream target of Akt [47].

Our results further indicate that molecules that are involved in Wnt signaling, including Tcf7L1, Frzb, and Wntless, were upregulated in DC-tumor fusions. Additionally, WISP-1 and NRCAM, targets of the canonical Wnt pathway, were upregulated 12- and 148.7-fold, respectively. In the canonical Wnt pathway, activation of Wnt receptors leads to stabilization and import of *β*-catenin into the nucleus where *β*-catenin associates with T-cell factor/lymphoid enhancer factor (TCF/LEF) and activates target genes. However, treatment of DC-tumor fusions with JW74, a specific inhibitor of the canonical Wnt pathway, had no impact on IL-12 secretion by these cells. On the other hand, it is conceivable that the mTOR pathway was activated through the noncanonical Wnt/Ca^2+^ pathway, Wnt-dependent activation of PKA (protein kinase A) and CREB (cAMP response element-binding protein), or mTOR activation via Wnt-mediated inhibition of glycogen synthase kinase 3. Alternatively, we cannot exclude that the PI3K/Akt/mTOR pathway was activated independent of Wnt signaling.

The PI3K/Akt/mTOR pathway has been shown to play a critical role in cell proliferation, survival, and metastasis of cancer cells [48], and we observed that inhibition of the PI3K/Akt* and inhibition* of the mTOR pathway enhanced the immune-stimulatory capacity of DC-tumor fusions through induction of bioactive IL-12p70 secretion. The fact that combined inhibition of PI3K and mTOR signaling did not further improve IL-12p70 secretion by DC-tumor fusions may indicate that inhibition acted on the same signaling pathway, likely to involve p70^S6K^ as has been described previously [49].

In sum, we conclude that combining PI3K/Akt/mTOR inhibition with DC-melanoma fusion cell-based cancer vaccination appears to be a promising strategy and warrants further studies* in vitro* and in animal models. Ultimately, this research may lead to the development of improved DC-fusion-based cancer vaccines with enhanced clinical impact.

## Abbreviations

Akt/PKB: Protein kinase B
CFSE: Carboxyfluorescein diacetate succinimidyl ester
CM: Complete media
DC: Dendritic cell
ELISA: Enzyme-linked immunosorbent assay
FACS: Fluorescence-assisted cell sorting
IFN: Interferon
IL: Interleukin
MEK: Mitogen extracellular signal-regulated kinase
mTOR: Mammalian target of rapamycin
LPS: Lipopolysaccharide
PI3K: Phosphatidylinositol 3-kinase
qPCR: Quantitative real-time PCR
Th: T helper.
